# Structural basis for PPARγ transactivation by endocrine-disrupting organotin compounds

**DOI:** 10.1038/srep08520

**Published:** 2015-02-17

**Authors:** Shusaku Harada, Youhei Hiromori, Shota Nakamura, Kazuki Kawahara, Shunsuke Fukakusa, Takahiro Maruno, Masanori Noda, Susumu Uchiyama, Kiichi Fukui, Jun-ichi Nishikawa, Hisamitsu Nagase, Yuji Kobayashi, Takuya Yoshida, Tadayasu Ohkubo, Tsuyoshi Nakanishi

**Affiliations:** 1 Graduate School of Pharmaceutical Sciences, Osaka University, 1-6 Yamadaoka, Suita, Osaka 565-0871, Japan; 2 Laboratory of Hygienic Chemistry and Molecular Toxicology, Gifu Pharmaceutical University, 1-25-4 Daigaku-nishi, Gifu, Gifu, 501-1196, Japan; 3 Department of Pharmacy, College of Pharmacy, Kinjo Gakuin University, 2-1723 Omori, Moriyamaku, Nagoya, Aichi, 463-8521, Japan; 4 Research Institute for Microbial Diseases, Osaka University, Suita, Osaka 565-0871, Japan; 5 Department of Biotechnology, Graduate School of Engineering, Osaka University, 2-1 Yamadaoka, Suita, Osaka 565-0871, Japan; 6 Laboratory of Health Sciences, School of Pharmacy and Pharmaceutical Sciences, Mukogawa Women's Univerasity, 11-68 Kyuban-cho, Koshien, Nishinomiya, Hyogo, 663-8179, Japan

## Abstract

Organotin compounds such as triphenyltin (TPT) and tributyltin (TBT) act as endocrine disruptors through the peroxisome proliferator–activated receptor γ (PPARγ) signaling pathway. We recently found that TPT is a particularly strong agonist of PPARγ. To elucidate the mechanism underlying organotin-dependent PPARγ activation, we here analyzed the interactions of PPARγ ligand-binding domain (LBD) with TPT and TBT by using X-ray crystallography and mass spectroscopy in conjunction with cell-based activity assays. Crystal structures of PPARγ-LBD/TBT and PPARγ-LBD/TPT complexes were determined at 1.95 Å and 1.89 Å, respectively. Specific binding of organotins is achieved through non-covalent ionic interactions between the sulfur atom of Cys285 and the tin atom. Comparisons of the determined structures suggest that the strong activity of TPT arises through interactions with helix 12 of LBD primarily via π-π interactions. Our findings elucidate the structural basis of PPARγ activation by TPT.

The peroxisome proliferator-activated receptors (PPARs), subtypes of which have been identified as α, γ, and δ, belong to the nuclear receptor superfamily and act as transcription factors to control the expression of target genes. PPARs form heterodimers with retinoid X receptor (RXR) and bind to specific regions on various genes^1^. It has been well established that PPARγ regulates the expression of the genes responsible for adipocyte differentiation. Furthermore, PPARγ has been found in trophoblasts, where it serves as an essential regulator of placental differentiation and has other endocrine functions, including the production of human chorionic gonadotropin (hCG) and in steroidogenesis^2^,^3^,^4^.

Organotin compounds have been widely used as antifouling biocides for ships, agricultural fungicides, and so on^5^,^6^. However, their widespread use has deleteriously affected marine ecosystems. At very low concentrations, organotin compounds such as triphenyltin (TPT) and tributyltin (TBT) induce “imposex”, which is the masculinization of female gastropod mollusks^7^. Hence these tin compounds came to be known as endocrine-disrupting chemicals. In mammals, organotins also have various undesirable effects on immune mechanisms and metabolic activity^5^,^8^. We previously investigated the effects of organotins on PPARγ and showed that: (1) TPT and TBT at nanomolar concentrations enhance hCG production in human choriocarcinoma cells and stimulate adipocyte differentiation; (2) the endocrine disruptive action of organotins is mediated through the PPARγ-dependent pathway; (3) TPT has considerably stronger agonistic activity toward PPARγ than does TBT; and (4) tri-alkyl and aromatic tin compounds have stronger agonist activities than do tetra-, di- and monosubstituted compounds^9^,^10^,^11^,^12^,^13^,^14^.

Recently, a crystal structural analysis of the RXRα/TBT complex was performed that provided insights into how TBT activates the RXRα-PPARγ signaling pathway^15^; transactivation by organotins was attributed to RXRα, not to PPARγ, because of the weak agonistic activity of TBT toward PPARγ. In addition, the study^15^ reported that organotin compounds employ a covalent interaction between the tin atom and a particular cysteine residue (Cys432) located in helix 11 (H11) of RXRα. Stabilization of this secondary structural element is believed to be essential to modulate transcriptional activity. Due to the absence of a cysteine residue at the corresponding position in PPARγ, the authors of the study^15^ concluded that the binding of organotin compounds to PPARγ alone does not allow efficient transactivation. Therefore, our finding that TPT acts as a powerful PPARγ agonist^9^ suggests the need to clarify the structural basis for PPARγ transactivation by tin compounds in order to better understand the mechanism of RXR-PPARγ signaling via these compounds.

Since the first structural determination of the PPARγ ligand-binding domain (LBD) was reported by using X-ray crystallography^16^, multiple structures in both the apo and liganded forms have been determined. From these studies, activation mechanisms induced by various well-known PPARγ agonists, including thiazolidinediones (TZD) such as rosiglitazone, have been discovered. The binding of the agonist to the ligand-binding pocket of LBD causes its helix 12 (H12) to form an active conformation that promotes the recruitment of a transcriptional coactivator. However, the mechanism of transactivation by tin compounds with various substituted organic groups, which are distinct from well-known PPARγ agonists with respect to their structural and chemical features (see Supplementary Fig. S1 online), is still poorly understood and cannot be inferred based on previously known structures. Therefore, to elucidate the mechanism, here we determined the three-dimensional structures of PPARγ-LBD in complexes with TPT and TBT, respectively, and characterized these complexes by using mass spectrometry (MS) and biological activity assays.

## Results

### Structural determination of the PPARγ complexes

**Figure 1:**
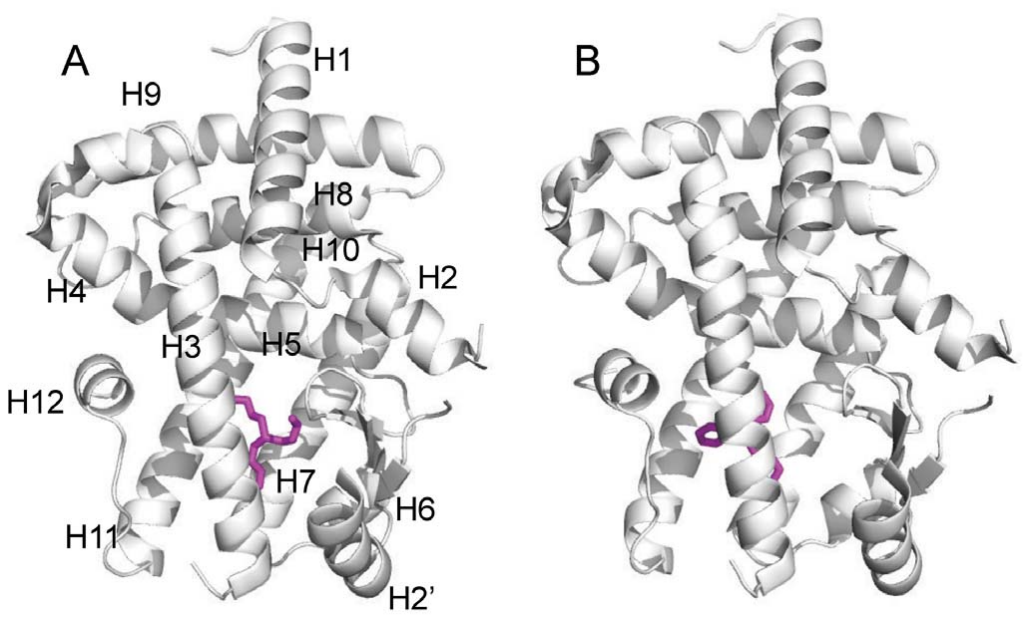
Structures of the PPARγ-LBD complex with TBT (A) and TPT (B).

Crystals of PPARγ-LBD in complex with TPT and TBT, respectively, were obtained by co-crystallization. They belong to the same space group *P*2_1_ with similar cell dimensions. The structures determined at 1.95 Å (PPARγ-LBD/TBT) and 1.89 Å (PPARγ-LBD/TPT) resolutions (Table 1) were revealed to have the typical nuclear receptor fold that comprises an α-helical sandwich fold (12 helices) with a four-stranded β-sheet. In an asymmetric unit, two PPARγ-LBD molecules were found. These structural data have been deposited in the Protein Data Bank database under the accession numbers 3WJ4 and 3WJ5. Both crystals contained two LBDs (chain A and B) in the asymmetric unit, where each LBD was bound to one ligand. The structure of chain A assumed an “active” conformation that was found in the PPARγ-LBD/agonist/co-activator peptide complex (PDB No. 2PRG)^16^, where helix 12 (H12) of LBD exists in a position suitable to interact with the agonist and to form the binding site of the co-activator. By contrast, in the other molecule (chain B), H12 was displaced from the ligand-binding pocket, preventing the co-activator from binding to the LBD, possibly due to extensive interactions with symmetry-related neighboring molecules in the crystal (see Supplementary Fig. S2 online). Because PPARγ transactivation is induced by the “active” conformation of H12, we hereafter focused on the structure of chain A (Figure 1).

**Figure 2:**
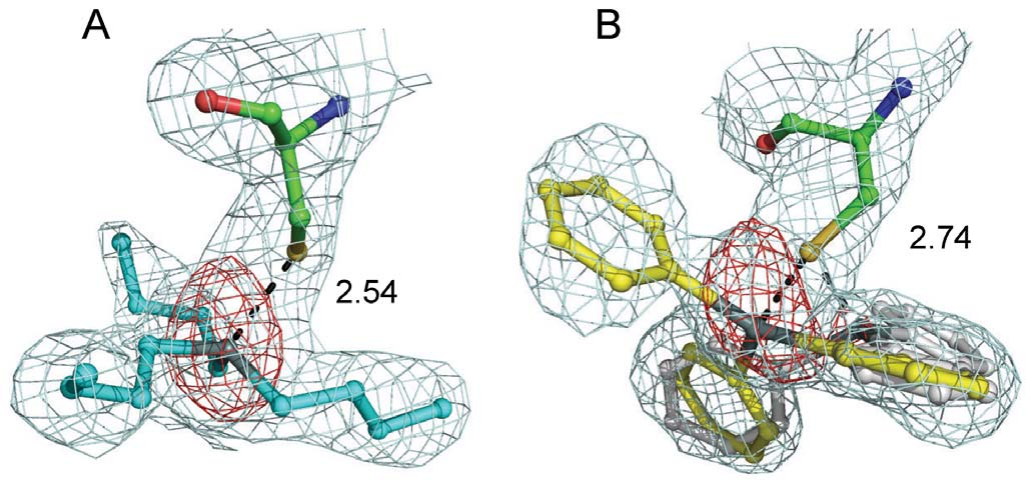
The organotins ((A): TBT, (B): TPT) and Cys285 in the ligand-binding pocket of PPARγ. Anomalous difference electron density maps contoured at 3.5σ (red) indicate the position of the tin atom and omit *2F_O_–F_C_* electron density maps contoured at 0.5σ (cyan) indicate the geometry of the aliphatic or aromatic chain. Distances between the tin and sulfur atoms are indicated. In panel (B), the major and minor conformations of TPT are shown in yellow and gray, respectively.

The exact positions of the ligands were determined by using an anomalous difference map derived from the tin anomalous signals (Figure 2). Although the structural analyses showed ligands with relatively low electron densities (Figure 2) and large B factors (Table 1), clear anomalous signals corresponding to tin atoms were found in close proximity to the sulfur atom of Cys285, which exists in the central region of the ligand-binding pocket. This finding suggests that specific interactions occur between the tin and sulfur atoms. In the TBT complex, only one anomalous peak was identified at the side of the β-strand (B3), at a distance of 2.54 Å from the sulfur atom of Cys285. In contrast, the TPT complex had a major (16σ) and a minor (5σ) anomalous peak around Cys285, which were associated with two alternative binding sites for TPT. Refinements provided the model with the occupancy of TPT molecule at the major site is 80%. Intriguingly, despite the structural similarities of the ligands, the major TPT complex was close to H12, which was on the opposite side of Cys285 from the position of TBT. The distance between the tin atom and the sulfur atom of Cys285 was 2.74 Å (Figure 2). For both complexes, the Sn-S lengths were longer than the sum of the covalent radii (2.42 Å) and equal to, or slightly longer than, the sum of the ionic radii (2.54 Å) of tin and sulfur, suggesting that the Sn-S bond is non-covalent and ionic rather than covalent, as previously observed in the crystal structure of the RXRα/TBT complex.

**Figure 3:**
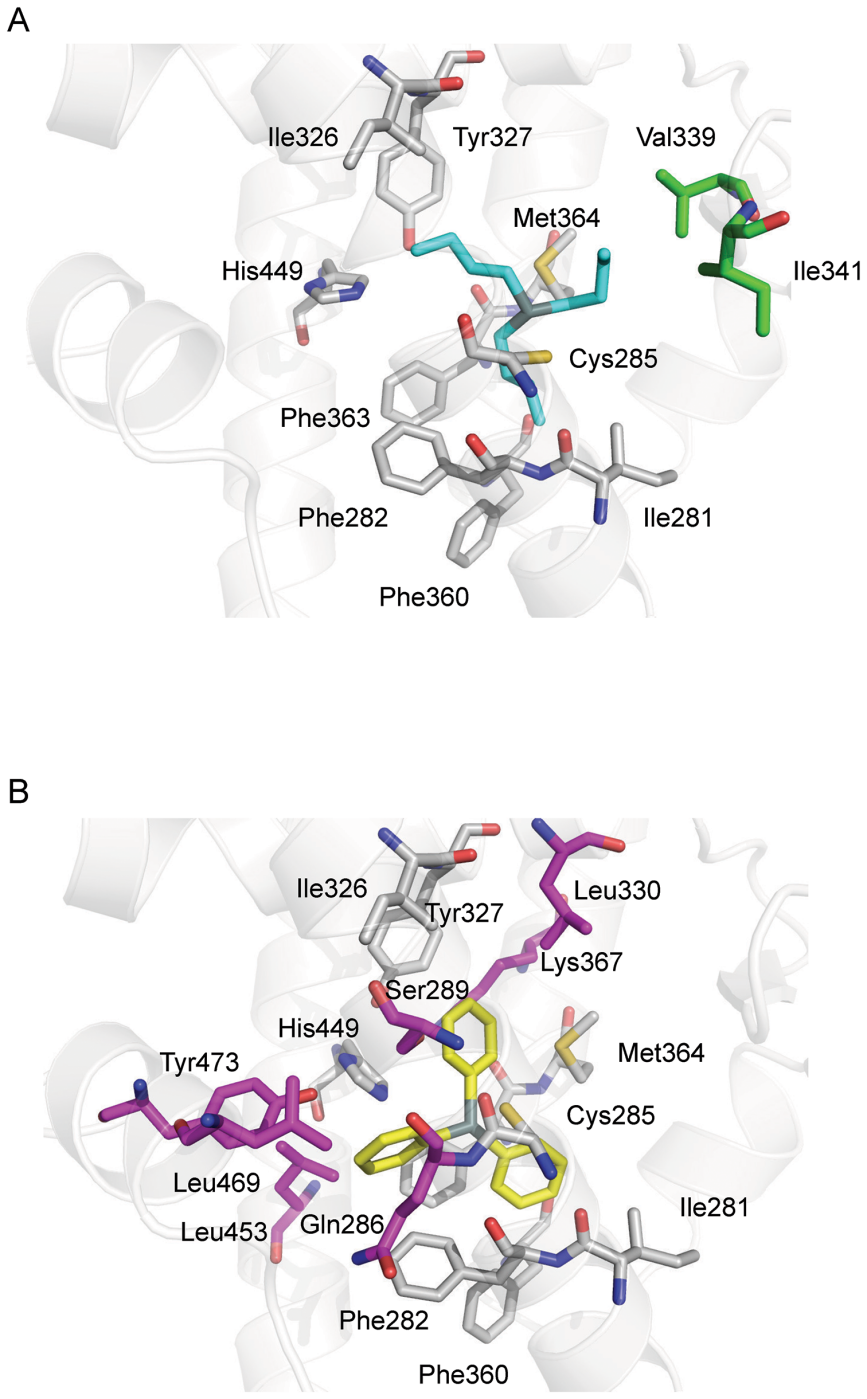
Interactions of PPARγ-LBD with TBT (A) or TPT (B). Ligands are shown as cyan and yellow sticks. Ligand-interacting residues, which are close (within 4.2 Å) to the ligands, are also shown. Common interacting residues for both ligands are shown as gray sticks. Interacting residues for TBT or TPT only are show in green or pink, respectively.

The PPARγ ligand-binding pocket is surrounded by secondary elements, B3, H3, H5, H7, H11, and H12. Cys285 in H3 serves an anchor for the tin atom to interact with the residues lining the pocket, which are predominantly hydrophobic in character, by using the alkylic or aromatic moieties of the organotins. Both ligands share common interacting residues, namely Ile281 (H3), Phe282 (H3), Ile326 (H5), Tyr327 (H5), Phe360 (H7), Phe363 (H7), Met364 (H7), and His449 (H11). Besides these residues, TPT also interacts with Gln286 (H3), Ser289 (H3), Leu330 (H5), Lys367 (H7), Leu453 (H11), Leu469 (H12), and Tyr473 (H12). In contrast, TBT interacts with Val339 (B3) and Ile341 (B3) (Figure 3).

### Activity of tin compounds as PPARγ agonists

**Figure 4:**
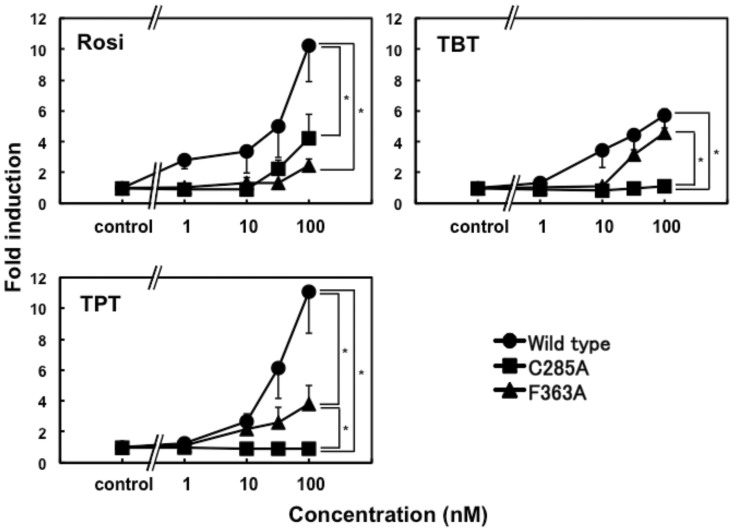
Cell-based transcriptional activation assay of rosiglitazone, TPT, and TBT on wild-type, C285A, and F363A mutants of PPARγ. Data are expressed relative to the levels of vehicle-treated cells; these levels were set to 1. Results are expressed as means ± 1 S.D. of three independent cultures. *P < 0.05 indicates values significantly different between 2 groups analyzed by using 2-way ANOVA.

To evaluate the functional potency of tin compounds as PPARγ agonists, we performed reporter assays using the PPARγ-specific GAL4-luc system, where wild-type PPARγ-LBD is fused to the GAL4 DNA-binding domain and a luciferase reporter gene is under the control of GAL4 binding elements (Figure 4). TPT (100 nM) enhanced the transactivation function of PPARγ by 11-fold, and this level of activation was comparable to that of rosiglitazone, a representative full agonist for PPARγ. However, although lower concentrations of TPT and TBT provided similar responses to that of rosiglitazone, 100 nM TBT showed only half the level of activation achieved with rosiglitazone and TPT. These results indicate that, despite similar structural and chemical features, TPT and TBT differ in their PPARγ transactivation and/or binding.

To verify the involvement of the π-π interaction in the full-agonistic activity of TPT, we substituted Phe363 of PPARγ with alanine and carried out the cell-based assay. In the case of TPT, the transcriptional activity of the mutant was about three-fold lower than that of wild-type, whereas no significant difference in activity was observed for TBT upon the introduction of the mutation (Figure 4).

### Characterization of organotin binding to PPARγ Cys285

**Figure 5:**
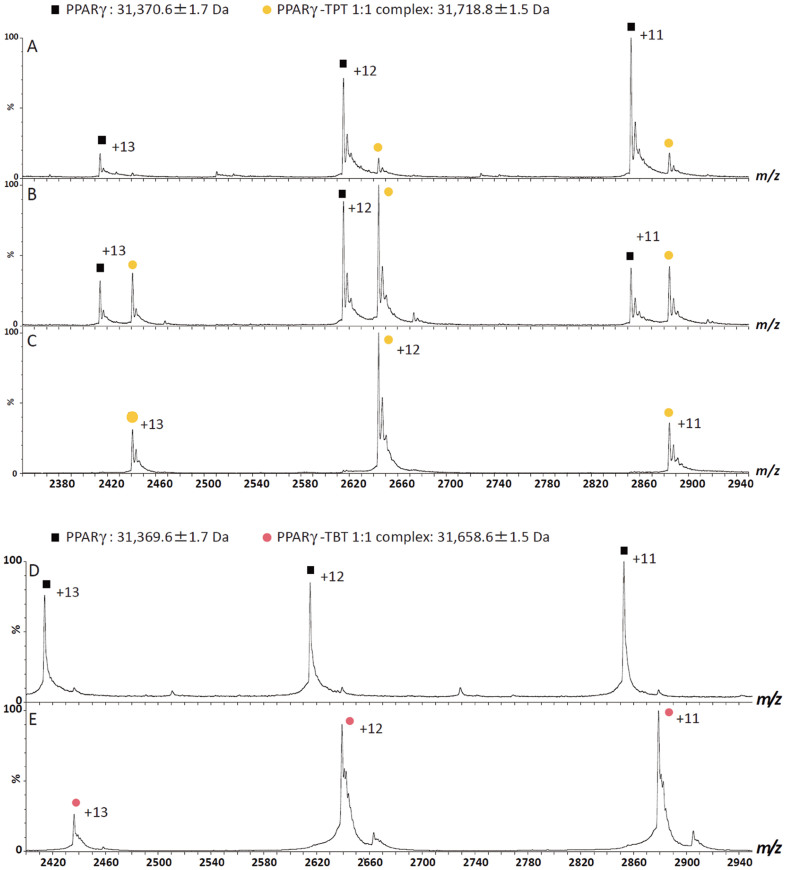
Mass spectrometry of the PPARγ-LBD complex with TPT (A–C) or TBT (D, E) under non-denaturing conditions. Mass spectra show that PPARγ forms a complex with TPT (C) or TBT (E) in a 1:1 molar ratio. Mass patterns after the addition of aliquots of formic acid (A, D = 3%, B = 1%) to the complex indicate that the dissociation of the interaction is caused by the unfolding of PPARγ-LBD.

The PPARγ mutant with its Cys285 replaced to alanine was not activated by TBT and TPT (Figure 4). Thus, as was previously shown for RXR^15^, the specific interaction between the cysteine residue and the tin atom is essential for the activation of PPARγ by organotins. However, our structural data suggested that the Sn-S bonds in PPARγ complexes are ionic in nature, in contrast to the covalent bond reported for the RXRα-LBD/TBT complex^15^. To clarify whether complex formation of PPARγ-LBD with tin compounds is mediated through covalent or non-covalent bonding, we performed MS analysis under non-denaturing conditions for PPARγ-LBD complexes with TPT or TBT (Figure 5). The results indicated that the complexes of PPARγ with these organotins formed in 1:1 molar ratios (Figure 5C and 5E). No free PPARγ-LBD was detected in these spectra and even under highly stringent ionization conditions of up to 190 V of sample cone voltage (*V*c), the complex peaks were not disrupted, indicating that the complexes for both cases were highly stable.

On the other hand, the addition of aliquots of formic acid to the mixtures, which should induce PPARγ-LBD unfolding, resulted in different MS patterns (Figure 5A, B, and D). The newly emerged ion series under the partially (Figure 5B) or fully (Figure 5A and 5D) PPARγ-LBD unfolded conditions yielded the molecular mass of free PPARγ-LBD (31,370.6 Da), indicating the dissociation of TPT or TBT from PPARγ-LBD upon the acid-induced unfolding of PPARγ-LBD in the mixture. Furthermore, the addition of a sufficient amount of formic acid to the mixture of PPARγ-LBD and TPT led to the complete disappearance of the peaks for the PPARγ-LBD/TPT complex, whereas, in addition to the peaks of unfolded PPARγ-LBD, the peak of dissociated TPT was consistently observed as a singly charged species with a molecular mass of 350.1 Da (see Supplementary Fig. S3 online). In the previous study^15^, they proposed that TBT is connected to RXRα through a “covalent bond” involving the sulfur atom of RXRα Cys432 based on the crystal structure and on MS results that showed no disruption of the RXRα-LBD/TBT complex even when highly stringent parameters (*V*c: 190 V) were applied. Similar to their results, in our current study, the PPARγ-LBD/TPT and PPARγ-LBD/TBT complexes were retained even under highly stringent conditions (*V*c: 190 V). However, our current structural study clearly showed the absence of a covalent bond in the PPARγ-LBD/TPT and PPARγ-LBD/TBT complexes. Given that little or no dissociation of protein metal complexes in the gas phase has been observed in the mass spectra of metal complexes^17^,^18^ and that hydrogen bonding and electrostatic interactions are maintained to a greater extent than are hydrophobic (van der Waals) interactions^19^, the retention of the non-covalent interactions of nuclear receptors/organotin complexes under non-denaturing MS conditions with highly stringent parameters is not unexpected. We also performed MS analysis under fully denaturing conditions in which a covalently-bound 15-deoxy-Δ12,14-prostaglandin J2 (15d-PGJ2)^20^ did not dissociate from PPARγ-LBD while a non-covalent ligand, rosiglitazone, did. The results demonstrated that the complexes of PPARγ with organotins dissociates in the denaturing conditions (see Supplementary Fig. S4 online).

Therefore, we re-investigated the binding mode of the RXRα-LBD/TBT interaction by performing an MS analysis of the RXRα-LBD/TBT mixture. Consistent with the previous report^15^, peaks of RXRα-LBD/TBT complex with similar charge states could be observed without disruption of the complex formation. However, the bound TBT was dissociated with ease when RXRα was partially or completely unfolded by the addition of acetonitrile or formic acid to the solution (see Supplementary Fig. S5 online). These results indicate that TBT is bound to RXRα via a non-covalent interaction.

Taken together, we conclude that TPT and TBT bind to PPARγ principally via a non-covalent, ionic bond between the tin atom and Cys285 that requires the correct folding of PPARγ-LBD, which provides appropriate electrostatic and van der Waals interactions.

## Discussion

Recent pharmacological studies classify agonists of nuclear receptors as full or partial agonists, depending on their transcriptional activities. The difference between these two types of agonist can be explained in terms of the structural features of PPARγ-LBD/agonist complexes and whether they use the H12-mediated or non-H12-mediated mechanism. The full agonists directly stabilize H12, allowing it to dock against H3 and H11^16^,^21^. The coactivator-binding interface is configured by this conformational change. In contrast, the partial agonists have no direct contact with H12, predominantly interacting with residues on H3 and B3^22^. This classification also provides a means to distinguish between the differences in activity between the tin compounds (Figure 4). TPT, a full agonist as shown by cell-based assays (Figure 4), has hydrophobic interactions with Leu469 and Tyr473, stabilizing H12. In contrast, the hydrophobic interactions of TBT with the side chains of Val339 and Ile341, located in B3, displace TBT from H12. This structural feature of the TBT complex suggests some limited agonistic activity for TBT, as observed in the cell-based assay (Figure 4).

**Figure 6:**
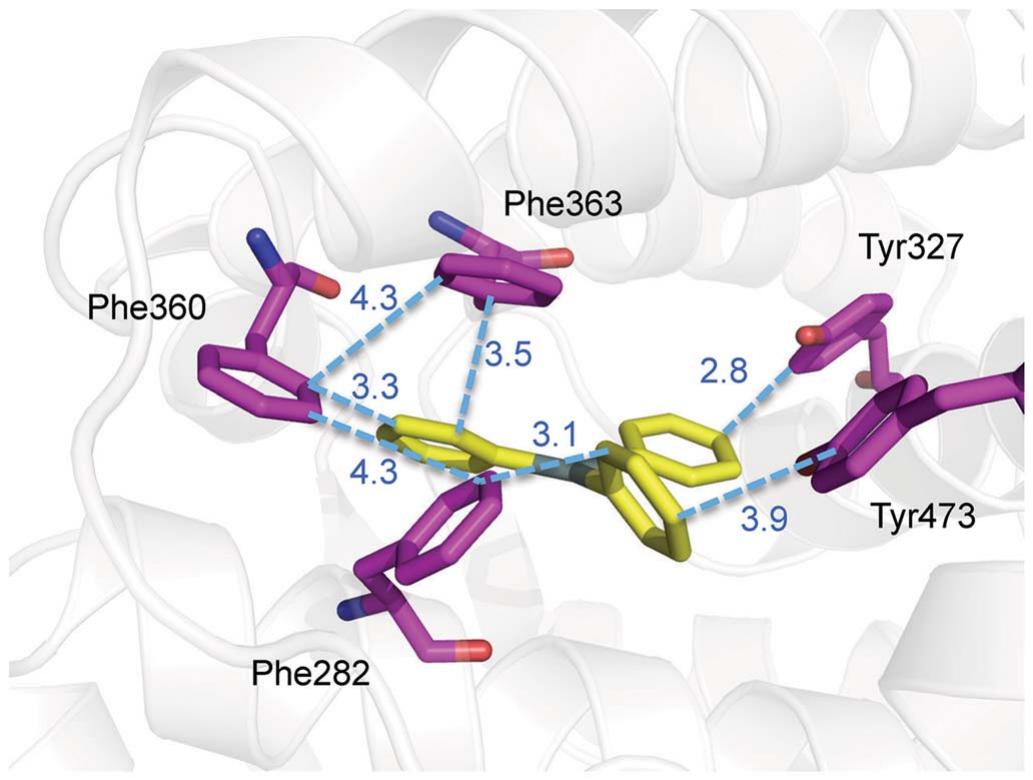
The π-π interactions in the PPARγ-LBD/TPT complex. The distances between the nearest neighbor carbon atoms of the aromatic rings are indicated.

In both complexes, the protein backbones have almost the same conformation, and the ligand volumes (TBT, 233 Å^3^; TPT, 245 Å^3^) are very similar^15^. Thus, it is likely that both organotin compounds would interact with PPARγ-LBD in the same manner and show similar transactivation activity. However, we found that they occupy different spatial positions within the ligand-binding pocket that result in different levels of activation. Of note, organotin compounds are anchored to the Cys285 of PPARγ by an ionic bond, which does not impose strict angular or length constraints, unlike covalent bonds. Moreover, PPARγ-LBD has a relatively large binding pocket, which can accommodate a diverse array of ligands^16^. Thus, TPT and TBT can optimally adapt their positions depending on their specific interactions with surrounding residues in the ligand-binding pocket, even though their ligand volumes are almost the same. The origins of positional preference are explained based on high-resolution structural analyses of the ligand-binding pocket, in which the side chains of Phe and Tyr form a cluster on the side of H12. The phenyl rings of TPT make it possible to form a network of π-π interactions, as shown in Figure 6, which stabilizes the active conformation of H12 and results in full agonistic activity. It is well known that π-π interactions can have a significant influence on protein–ligand interactions^23^. In a previous report^10^, we showed that TPT is powerful agonist for PPARγ but not for the other PPAR subtypes, α and δ, which have an isoleucine at the position corresponding to Phe363. The mechanism of subtype selectivity responsible for the presence of the π-π interaction at Phe363 has been proposed for benzenesulfonamide derivatives, which are selective PPARγ agonists^24^. In fact, although the transactivation of the F363A mutant of PPARγ by TPT was significant lower than that of wild-type, no difference in activity was observed for TBT (Figure 4). These results indicate that the π-π interactions contribute to the high transcriptional activity of TPT for PPARγ.

In contrast to TPT, TBT had no direct contact with H12 as shown in Figure 3A. This observation is consistent with previous findings where TBT behaved as a weak agonist against PPARγ and acted mainly on RXRα^15^. Although no direct interaction between the organotin and H12 was observed in the case of the RXRα/TBT complex, a specific interaction between the tin atom and Cys432 in H11 of RXRα might stabilize the helix 12. However, Cys285 of PPARγ, which is located at H3, offers an anchoring point to the organotin but is not sufficient to position the ligand such that it can support the active conformation of H12 (see Supplementary Fig. S6 online).

In conclusion, here we show the structural basis for the strong activation of PPARγ by TPT. We previously showed that hCG secretion in human choriocarcinoma cells, which is upregulated by RXR-PPARγ signaling pathways, is powerfully induced by phenyltin compounds, relative to butyltin compounds^9^,^12^,^13^ and concluded that the differences in toxicological response caused by these organotins depended on their potencies as agonists for PPARγ and RXR^9^. Our current observations show that the mode of action of organotin compounds, via RXR-PPARγ signaling pathways, is strongly influenced by their chemical structures.

## Methods

### Cell culture

Cells of the human choriocarcinoma cell line JEG-3 (ATCC No. HTB-36) were obtained from ATCC (Manassas, VA). JEG-3 cells were cultured in MEM with 2 mM L-glutamine, 0.1 mM MEM nonessential amino acid solution (Invitrogen, Carlsbad, CA), and 10% fetal calf serum (FCS). The cells were maintained at 37°C in a humidified atmosphere containing 5% CO_2_.

### Plasmid construction

Full-length cDNA of human PPARγ was amplified by RT-PCR using mRNA from JEG-3 cells. For the PPARγ transactivation assay, the amplified hPPARγ fragment was cloned into the pM vector (Clontech, Mountain View, CA). The resulting GAL4 DNA-binding domain- (DBD) fused hPPARγ expression vector was termed pM-hPPARγ. pM-hPPARγ mutant constructs, carrying a Cys285 or Phe363 to Ala mutation, were generated by site-directed mutagenesis of the pM-hPPARγ plasmid using the PrimeSTAR mutagenesis basal kit (Takara Bio, Shiga, Japan). The sequences of the mutagenic primers are shown in Supplementary Table S1, online.

### PPARγ transactivation assay

The responsiveness of PPARγ to organotin compounds was measured by using a chimeric receptor consisting of the GAL4-DBD and PPARγ, pM-hPPARγ, with a luciferase (LUC) reporter system, p4×UAS-tk-luc, which is a LUC reporter construct containing four copies of the GAL4 binding site [upstream activating sequence (UAS) of GAL4] followed by the thymidine kinase promoter^9^,^10^. Transient transfection assays were performed in JEG-3 cells with Lipofectamine reagent (Invitrogen). The cells (3 × 10^4^) were seeded in 24-well plates 24 h before transfection with pM-hPPARγ and p4×UAS-tk-luc. At 24 h after transfection, compounds in 0.1% DMSO were added to the cells, which were then cultured in medium supplemented with 1% charcoal-stripped FCS. The cells were harvested 24 h later, and extracts were assayed for firefly LUC activity. To normalize firefly LUC activity, the Renilla LUC control reporter construct pGL 4.74-TK (Promega, Madison, WI) was co-transfected as an internal standard. The results are expressed as the average of measurements of at least quadruplicate samples. Data from the cell-based transcriptional activation assay were analyzed by using two-way ANOVA, with multiple comparisons obtained with the Tukey-HSD test. A *P* value of <0.05 was used to indicate statistical significance. All statistical analyses were performed with SPSS software (Chicago, IL).

### Protein expression and purification

The human PPARγ-LBD (residues 202–477) was cloned into the pGEX-6P-3 vector. *E. coli* Rosetta (DE3) cells (Novagen) transformed by the plasmid were grown at 37°C in LB medium containing 20 μg/ml chloramphenicol and 50 μg/ml ampicillin to A_600_ = 0.6, and were induced by the addition of IPTG to a final concentration of 0.1 mM. Then, the cells were grown for 12–14 h at 10°C. Harvested cells were lysed by sonication. After the supernatant was applied to a GSTrap HP column (GE Healthcare), 50 mM Tris-HCl (pH 7.5), 150 mM NaCl, 1 mM EDTA, and 1 mM DTT with 100 U PreScission protease (GE Healthcare) was applied to same column, to remove the GST tag. PPARγ-LBD was eluted with the same buffer and dialyzed against 20 mM Tris-HCl (pH 8.0). Then PPARγ-LBD was applied to a HiTrap Q HP column (GE Healthcare) and eluted with an NaCl gradient (0.01–0.5 M). The fractions containing PPARγ-LBD were pooled and dialyzed against 20 mM Tris-HCl (pH 7.5), 100 mM NaCl, 5 mM EDTA, and 2 mM DTT. PPARγ-LBD was concentrated to 5 mg/mL by using Amicon Ultra 15 concentrator (Millipore).

### Crystallization, data collection, and structure determination

We performed co-crystallization with organotin compounds and PPARγ-LBD by using the sitting-drop vapor-diffusion method at 277 K. Because the organotin compounds have poor aqueous solubility, an excess of each compound was added as powder to the protein solution and incubated for several hours to obtain the PPARγ-LBD/organotin complex. Insoluble compound was removed before crystallization by centrifugation followed by filtration through a 0.2-μm membrane filter. Crystals were obtained from drops derived from 1 μL of protein solution (20 mM Tris–HCl, pH 7.5, 100 mM NaCl, 2 mM EDTA, 5 mM DTT) mixed with an equal volume of crystallization buffer (100 mM Tris-HCl, pH 8.5 160 mM CH_3_COONH_4_, 19%–23% PEG4000). Diffraction data were collected at 100 K on beamline 6A or 17A of Photon Factory, KEK (Tsukuba, Japan), and beamline BL38B1 of SPring-8 (Hyogo, Japan), and were indexed, integrated, and scaled by using HKL2000. All structures were solved by use of the molecular replacement method using MOLREP from the CCP4 suite with the previously reported PPARγ-LBD structure (PDB 1PRG) as an initial search model. Structural refinement and the addition of water molecules were performed by using Coot and REFMAC5. The final structures were checked and validated by MolProbity. The atomic coordinates were deposited in the Protein Data Bank (PDB) as entry code 3WJ4 and 3WJ5 for PPARγ-LBD/TBT and PPARγ-LBD/TPT, respectively. The statistics for the diffraction data collection and structural refinement are shown in Table 1.

### Mass spectrometry

The concentration of PPARγ-LBD was fixed at 1 mM, and an excess amount of TPT or TBT was added. After dilution with buffer (20 mM Tris and 150 mM NaCl pH 7.5) to 10 μM, sample solutions of PPARγ-LBD, PPARγ-LBD/TPT, and PPARγ-LBD/TBT were subjected to a buffer exchange with 150 mM ammonium acetate, pH 7.5, by passing them through mini gel filtration columns (BioRad) prior to analysis. All samples were analyzed by use of nanoflow electrospray using in-house capillaries prepared as described previously^25^. Samples were loaded into capillaries, and spectra were recorded on a modified Synapt HDMS mass spectrometer (Waters), which provides the molecular mass of a protein complex formed through a non-covalent interaction^26^. All mass spectra were calibrated against cesium iodide and analyzed by Mass Lynx software (Waters). Typical conditions included 2–3 μL of aqueous protein solution, capillary voltage of 1.1–1.7 kV, cone voltage of 190 V, and trap and transfer collision energy voltages of 30 and 10 V, respectively. The source pressure was maintained at 3 × 10^−2^ mbar.

## Author Contributions

S.H. prepared crystal samples and performed the X-ray analysis. Y.H. made constructs and performed the PPARγ transactivation assay. M.N., T.M., S.U. and K.F. performed the mass analysis. S.N., K.K. and S.F. provided technical assistance with crystallography. J.N. and H.N. provided technical assistance with cell biology. S.H., T.Y. and T.N. wrote the manuscript. Y.K. and T.O. edited the manuscript. T.N. and T.Y. conceived and designed the study.

## Supplementary Material

Supplementary Information Supplemental Information

## Figures and Tables

**Table 1:** Data collection and structural refinement statistics

	PPARγ-LBD/TBT	PPARγ-LBD/TPT
Data Collection		
Beamline	Photon factory BL-6A	Photon factory BL-17A
Wavelength (Å)	1.0000	0.9800
Space group	*P*2_1_	*P*2_1_
Cell dimensions		
*a*, *b*, *c* (Å)	56.34, 88.29, 57.51	56.52, 88.49, 57.94
*α*, *β*, *γ* (°)	90.00, 90.68, 90.00	90.00, 91.01, 90.00
Resolution range (Å)	50.00-1.95 (1.98-1.95)	50.00-1.89 (1.92-1.89)
*R*_merge_^b^	0.066 (0.352)	0.064 (0.372)
*<I/σI>*	43.25 (7.2)	44.12 (6.3)
Completeness (%)	100 (100)	99.8 (99.5)
Redundancy	7.6 (7.5)	7.6 (7.3)
Refinement		
Resolution (Å)	36.438-1.95 (2.00–1.95)	36.515-1.89 (1.94–1.89)
No. reflections	38942	43047
*R*_work_ (%)	0.194 (0.218)	0.203 (0.233)
*R*_free_ (%)	0.241 (0.270)	0.247 (0.247)
No. of non-H atoms		
Protein	4164	4116
Ligand	26	38
Water	224	223
Average B-factors		
Overall (Å^2^)	25.4	24.0
Protein (Å^2^)	25.0	23.7
Ligand (Å^2^)	33.2	48.2
Water (Å^2^)	26.2	26.2
R.M.S. deviations		
Bond length (Å)	0.017	0.017
Bond angles (°)	1.627	1.516
Ramachandran plot statistics		
Most favored (%)	98.62	98.81
Additional allowed (%)	1.38	0.99
Disallowed (%)	0.00	0.20

^a^Values in parentheses are for the highest resolution shell.

^b^*R*_merge_ = Σ|*I*_h_ − <*I*_h_>|/Σ*I*_h_, where <*I*_h_> is the average intensity of reflection h and symmetry-related reflections.

## Notes

Accession Codes: Structural data are available in the Protein Data Bank database under the accession numbers 3WJ4 (PPARγ-LBD/TBT complex) and 3WJ5 (PPARγ-LBD/TPT complex).
