# Supplementary material for: Structural basis for PPARγ transactivation by endocrine-disrupting organotin compounds
Source: Sci Rep. 2015 Feb 17;5:8520. doi: 10.1038/srep08520 (PMC4330522; doi:10.1038/srep08520)
Supplement: Supplementary Information — Supplemental Information [file srep08520-s1.pdf]

Supplemental Information

Structural basis for PPARγ transactivation by endocrine disrupting organotin compounds

Shusaku Harada[1], Youhei Hiromori[2,3], Shota Nakamura[4], Kazuki Kawahara[1], Shunsuke Fukakusa[1], Takahiro Maruno[5], Masanori Noda[5], Susumu Uchiyama[5], Kiichi Fukui[5], Jun-ichi Nishikawa[6], Hisamitsu Nagase[2], Yuji Kobayashi[5], Takuya Yoshida[1*], Tadayasu Ohkubo[1], Tsuyoshi Nakanishi[2*]

[1]Graduate School of Pharmaceutical Sciences, Osaka University, 1-6 Yamadaoka, Suita, Osaka 565-0871, Japan. [2]Laboratory of Hygienic Chemistry and Molecular Toxicology, Gifu Pharmaceutical University, 1-25-4 Daigaku-nishi, Gifu, Gifu, 501-1196, Japan. [3]Department of Pharmacy, College of Pharmacy, Kinjo Gakuin University, 2-1723 Omori, Moriyamaku, Nagoya, Aichi, 463-8521, Japan. [4]Research Institute for Microbial Diseases, Osaka University, Suita, Osaka 565-0871, Japan. [5]Department of Biotechnology, Graduate School of Engineering, Osaka University, 2-1 Yamadaoka, Suita, Osaka 565-0871, Japan. [6]Laboratory of Health Sciences, School of Pharmacy and Pharmaceutical Sciences, Mukogawa Women's Univerasity, 11-68 Kyuban-cho, Koshien, Nishinomiya, Hyogo, 663-8179, Japan.

[*] Correspondence and requests for materials should be addressed to T.N. (nakanishi@gifu-pu.ac.jp) or T.Y. (yo@phs.osaka-u.ac.jp)

Supporting Tables

Table S1 Data collection and structural refinement statistics

	PPARγ-LBD/TBT	PPARγ-LBD/TPT
**Data Collection**		
Beamline	Photon factory BL-6A	Photon factory BL-17A
Wavelength (Å)	1.0000	0.9800
Space group	$P2_1$	$P2_1$
Cell dimensions		
a, b, c (Å)	56.34, 88.29, 57.51	56.52, 88.49, 57.94
α, β, γ (°)	90.00, 90.68, 90.00	90.00, 91.01, 90.00
Resolution range (Å)	50.00-1.95（1.98-1.95）	50.00-1.89（1.92-1.89）
$R_{merge}{}^{b}$	0.066 (0.352)	0.064 (0.372)
$<I/\sigma I>$	43.25（7.2）	44.12（6.3）
Completeness (%)	100（100）	99.8（99.5）
Redundancy	7.6 (7.5)	7.6 (7.3)
**Refinement**		
Resolution (Å)	36.438-1.95 (2.00–1.95)	36.515-1.89 (1.94–1.89)
No. reflections	38942	43047
R_{work} (%)	0.194 (0.218)	0.203 (0.233)
R_{free} (%)	0.241 (0.270)	0.247 (0.247)
No. of non-H atoms		
Protein	4164	4116
Ligand	26	38
Water	224	223
Average B-factors		
Overall (Å^2)	25.4	24.0
Protein (Å^2)	25.0	23.7
Ligand (Å^2)	33.2	48.2
Water (Å^2)	26.2	26.2
R.M.S. deviations		
Bond length (Å)	0.017	0.017
Bond angles (°)	1.627	1.516
Ramachandran plot statistics		
Most favored (%)	98.62	98.81
Additional allowed (%)	1.38	0.99
Disallowed (%)	0.00	0.20

[a]Values in parentheses are for the highest resolution shell.

[b]$R_{merge} = \Sigma|I_h - <I_h>|/\Sigma I_h$, where $<I_h>$ is the average intensity of reflection h and symmetry-related reflections.

Table S2. Oligonucleotide primer sequences for site-directed mutagenesis

Mutations	Primer sequences	
	Sequence (5' to 3')	
C285A	Forward	CAG GGC **GCA** CAG TTT CGC TCC GTG GAG
	Reverse	GAA ACT G**TG C**GC CCT GAA AGA TGC GGA TGG
F363A	Forward	CTT TTG GTG AC**G CA**A TGG AGC CCA AGT TTG
	Reverse	CAT **TGC** GTC ACC AAA AGG CTT TCG CAG G

* Mutated nucleotide positions are shown in boldface type.

Supporting Figures

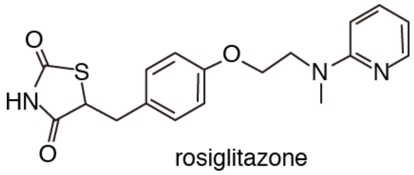

rosiglitazone

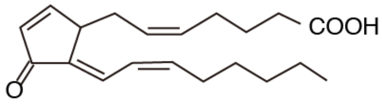

15d-PGJ2

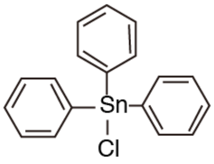

TPT

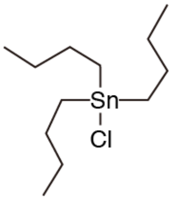

TBT

Fig. S1. Chemical structures of the ligands used in this study. Rosiglitazone is a representative of synthetic PPARγ full agonists. 15d-PGJ2 is an endogenous covalently-bound ligand of PPARγ. TPT (triphenyltin) and tributyltin (TBT) are organotin compounds.

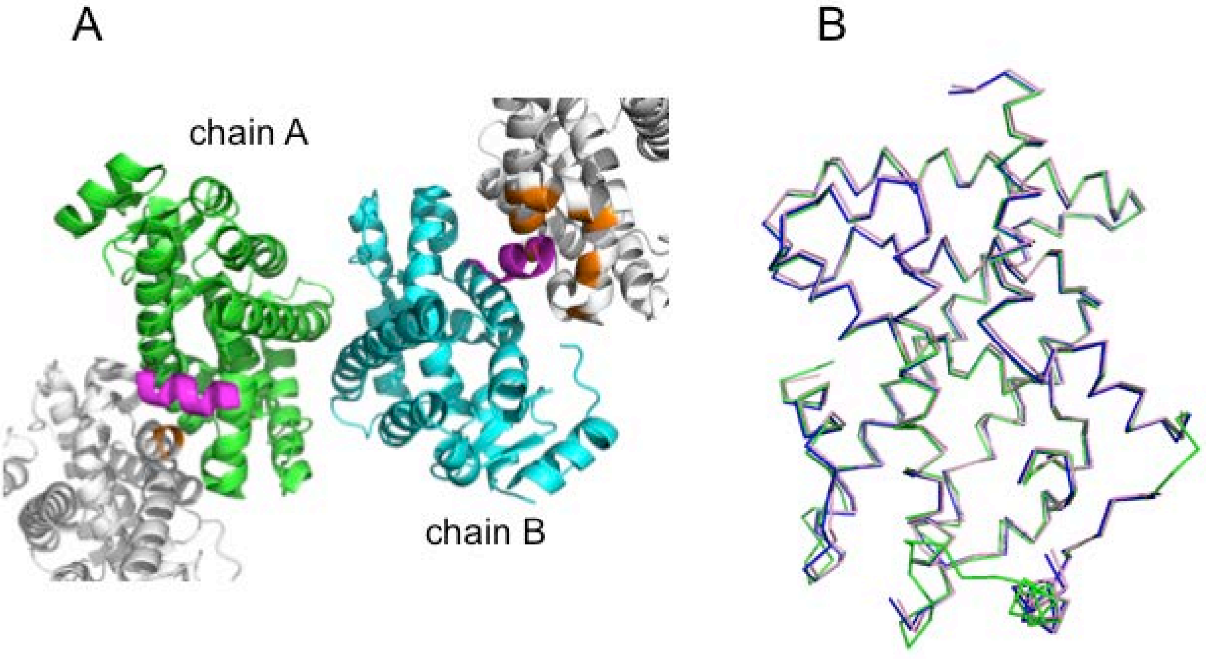

Fig. S2. (A) Crystal structure of PPARγ-LBD. The asymmetric unit contains two molecules of PPARγ-LBD, named chain A (green) and B (cyan). The residues of the neighboring symmetry mates (gray) that interact with helix12 (magenta in both chains), are shown in orange. (B) Superposition of Cα traces of PPARγ-LBD. The structure of chain A obtained in this study (blue, PPARγ-LBD–TBT; pink, PPARγ-LBD–TPT) closely resembles that of the PPARγ-LBD–agonist–co-activator peptide complex (green; PDB No. 2PRG, Nolte et al. 1998).

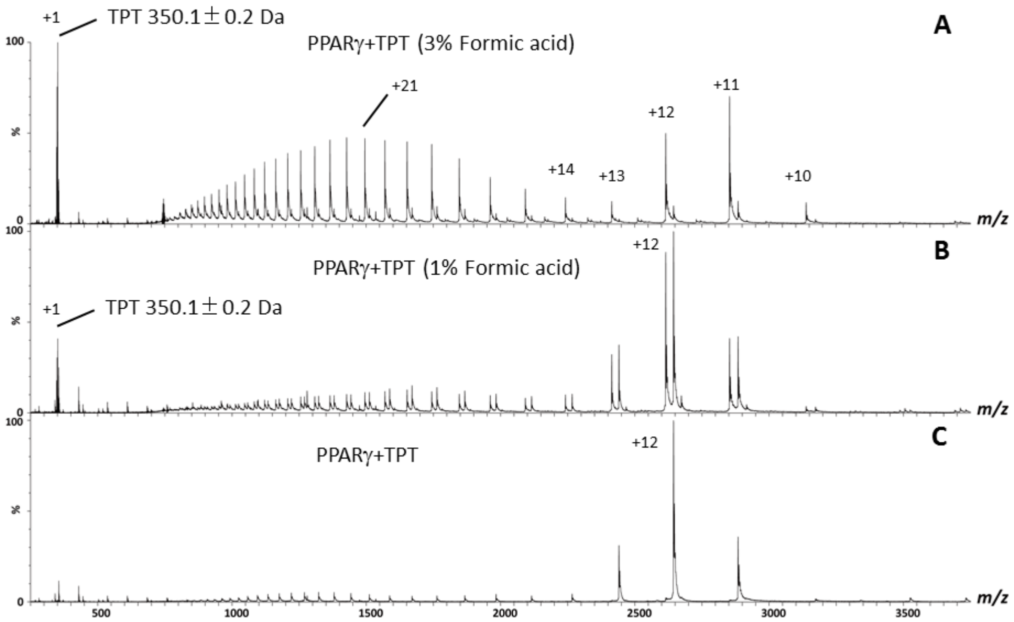

Fig. S3. Non-denaturing ESI-MS of TPT bound to PPARγ-LBD. The addition of aliquots of formic acid (A = 3%, B = 1%, C = 0%) to the PPARγ-LBD–TPT complex resulted in different MS patterns even under the same MS conditions. The results show that the gradual addition of formic acid to the PPARγ-TPT complex causes the dissociation of TPT, which is observed as a singly charged species with a molecular mass of 350.1 Da.

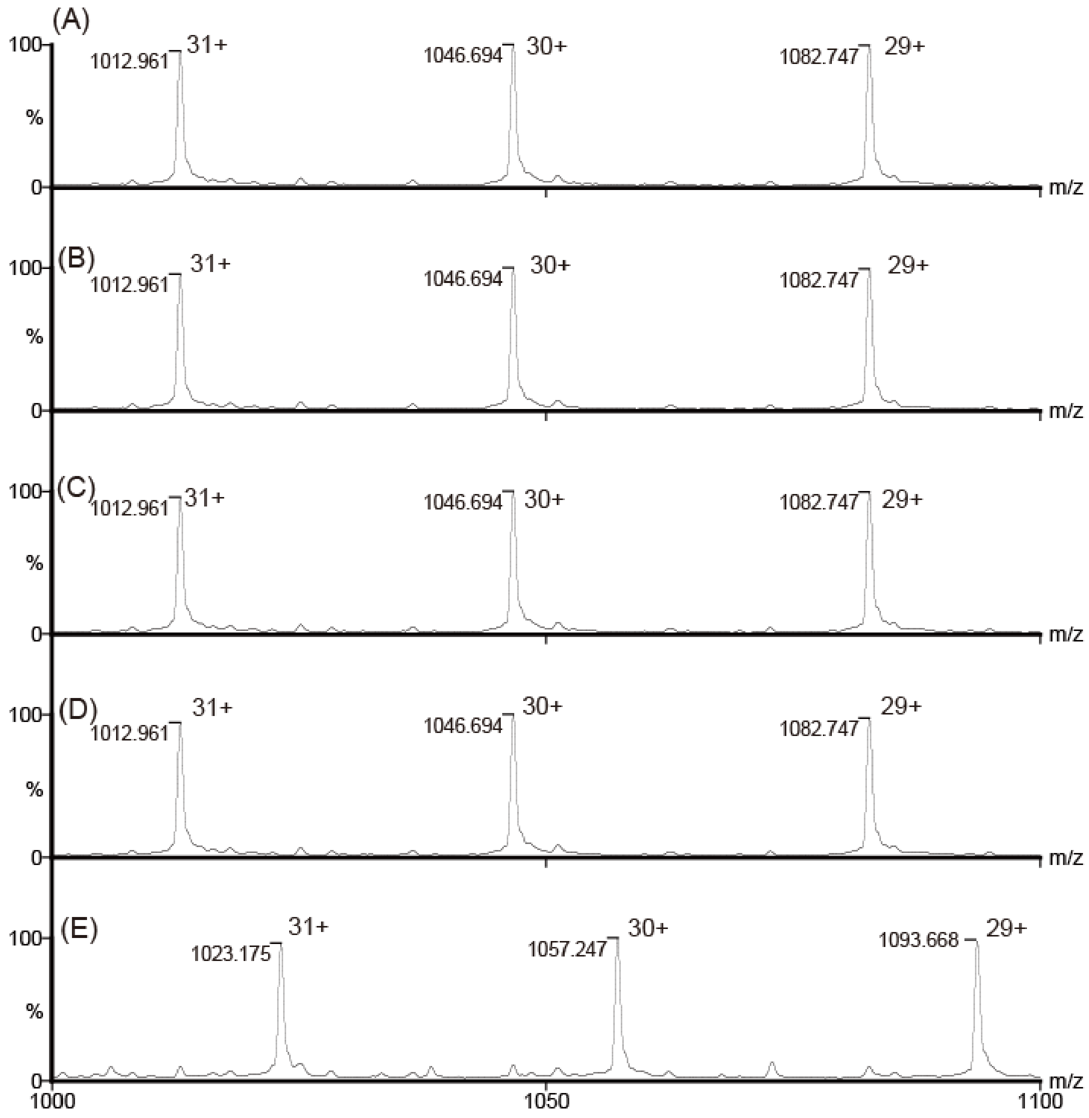

Fig. S4. Denaturing LC-ESI-MS spectra of PPARγ-LBD (A) and its complexes with TBT (B), TPT (C), rosiglitazone (D), and 15d-PGJ2 (E). Observed average mass values are 31370.4 Da (A-D) and 31687.0 Da (E). The increase in mass of PPARγ-LBD/15d-PGJ2 (+316.6 Da) indicates that covalent-bound 15d-PGJ2 (Calc: 316.4 Da) was not dissociated in the denaturing conditions.

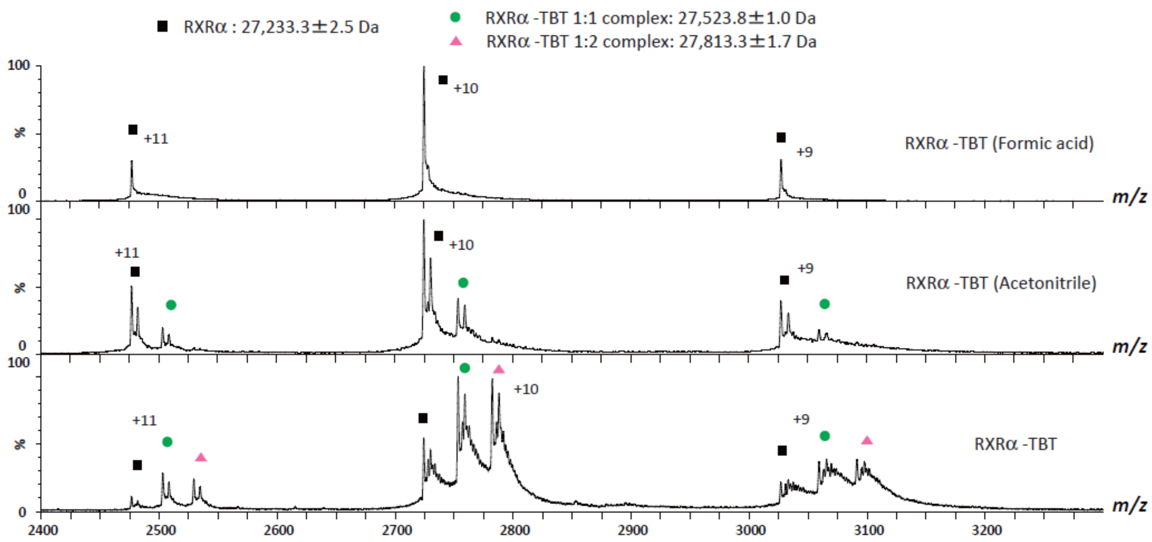

Fig. S5. Non-denaturing ESI-MS of TBT bound to RXRα-LBD. The addition of aliquots of 3% formic acid or acetonitrile to RXRα-LBD–TBT. TBT dissociates from RXRα under these conditions.

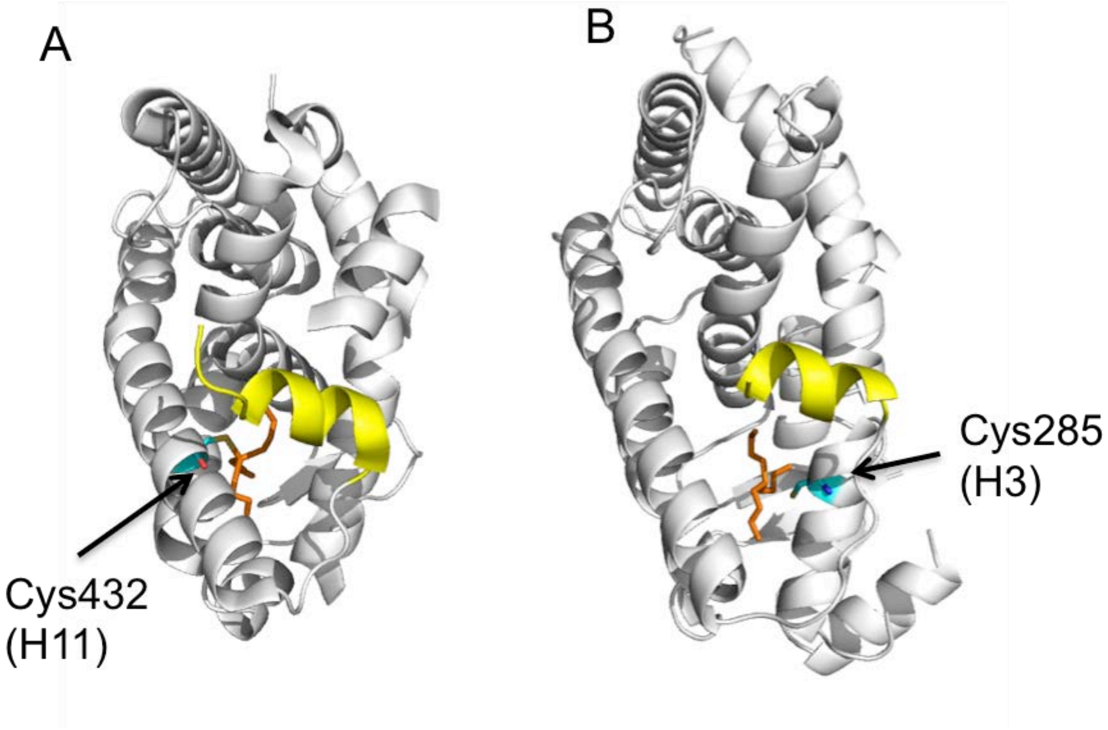

Fig S6. TBT-bound structure of the ligand-binding domain of RXRα (A) and PPARγ (B). Helix 12 is shown in yellow. The anchoring cysteine for TBT is colored in cyan. The TBT molecule is depicted as orange sticks.

Supporting Methods

Expression and purification of RXRα-LBD.

Human RXRα-LBD (residues 223–462) was cloned into vector pET-15b, which was used to transform *E. coli* BL21(DE3) cells. Transformants were grown at 37 °C in LB medium containing 50 μg/ml ampicillin to A_{600} = 0.6, and then induced by the addition of IPTG at a final concentration of 1 mM. The cells were then grown for an additional 4 h. Harvested cells were lysed by sonication. The supernatant was applied to a HisTrap HP column (GE Healthcare) equilibrated with buffer (50 mM Tris-HCl [pH 8.0], 500 mM NaCl). RXRα-LBD was eluted with the same buffer containing 125 mM imidazole and dialyzed against 20 mM Tris-HCl (pH 8.0). The His-tag was removed by proteolysis with thrombin. Finally, RXRα-LBD was purified by gel filtration on Superdex S200 and concentrated by using an Amicon Ultra 15 concentrator (Millipore).

Mass spectroscopy in denaturing condition.

LC-ESI-MS analyses were performed on an ACQUITY UPLC system (Waters, MA, USA) coupled to a SYNAPT G2 mass spectrometer (Waters, MA, USA). Prior to LC-ESI-MS analysis, PPARγ (50 μM) were incubated with TPT-Cl, TBT-Cl, rosiglitazone, and 15d-PGJ2 (500 μM each) for 3 hours. All samples (1 μg each) were injected onto a Waters MassPREP Micro Desalting column (60°C column temperature). Samples were eluted from the column with a 10 min gradient (5–90% B, 0.200 mL/min flow rate). Mobile phase A was 0.1% FA in H_2O. Mobile phase B was 0.1% FA in acetonitrile. The SYNAPT G2 mass spectrometer was run in positive ion, sensitivity mode with detection in the range of 100–5000 m/z. Source parameters were as follows: capillary voltage, 3.00 kV; sampling cone voltage, 40.0 V; source temperature, 150°C; desolvation temperature, 350°C; cone gas flow: 100 L/hr; desolvation gas flow, 800 L/hr. The mass spectrometry was calibrated with cesium iodide (Sigma-Aldrich Co. MO, USA), and lock mass were performed with [Glu1]-Fibrinopeptide B (Sigma-Aldrich Co. MO, USA). Molecular

mass was calculated by MassLynx software (version 4.1, Waters, MA, USA).
